# Nickel supported on nitrogen-doped carbon nanotubes as hydrogen oxidation reaction catalyst in alkaline electrolyte

**DOI:** 10.1038/ncomms10141

**Published:** 2016-01-14

**Authors:** Zhongbin Zhuang, Stephen A. Giles, Jie Zheng, Glen R. Jenness, Stavros Caratzoulas, Dionisios G. Vlachos, Yushan Yan

**Affiliations:** 1 State Key Lab of Organic-Inorganic Composites, Beijing University of Chemical Technology, Beijing 100029, China; 2 Department of Chemical and Biomolecular Engineering, University of Delaware, Newark, Delaware 19716, USA; 3 Center for Catalytic Science and Technology, University of Delaware, Newark, Delaware 19716, USA; 4 Catalysis Center for Energy Innovation, University of Delaware, Newark, Delaware 19716, USA

## Abstract

The development of a low-cost, high-performance platinum-group-metal-free hydroxide exchange membrane fuel cell is hindered by the lack of a hydrogen oxidation reaction catalyst at the anode. Here we report that a composite catalyst, nickel nanoparticles supported on nitrogen-doped carbon nanotubes, has hydrogen oxidation activity similar to platinum-group metals in alkaline electrolyte. Although nitrogen-doped carbon nanotubes are a very poor hydrogen oxidation catalyst, as a support, it increases the catalytic performance of nickel nanoparticles by a factor of 33 (mass activity) or 21 (exchange current density) relative to unsupported nickel nanoparticles. Density functional theory calculations indicate that the nitrogen-doped support stabilizes the nanoparticle against reconstruction, while nitrogen located at the edge of the nanoparticle tunes local adsorption sites by affecting the *d*-orbitals of nickel. Owing to its high activity and low cost, our catalyst shows significant potential for use in low-cost, high-performance fuel cells.

## Abstract (web summary)

Cheap and efficient hydrogen oxidation catalysts are needed for low cost hydroxide exchange membrane fuel cells. Here, the authors report that nickel nanoparticles supported on nitrogen doped carbon nanotubes have hydrogen oxidation activity similar to platinum-group-metals in alkaline electrolyte.

## Graphical abstract

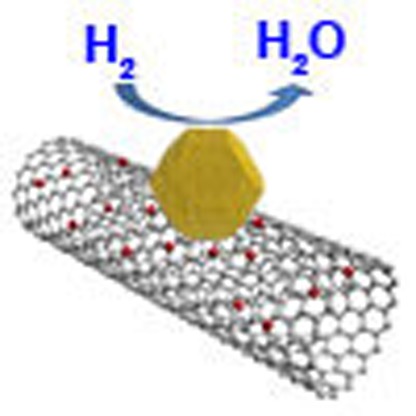

The hydrogen economy provides an efficient and environmentally friendly pathway to store and consume energy^1^,^2^. Fuel cells, especially the recently developed polymer electrolyte membrane fuel cells, are considered as the most promising device to convert the chemical energy of hydrogen to electricity^3^. The hydrogen fuel cells are based on two half-cell reactions: hydrogen oxidation reaction (HOR) at the anode and oxygen reduction reaction (ORR) at the cathode. To date, Pt is the most active catalyst for both HOR and ORR^4^. However, the commercialization of fuel cells is hindered by the high price of Pt (∼$50 g^−1^). Although the total content of platinum-group metals (PGMs) in the state-of-the-art proton exchange membrane fuel cell (PEMFC) stacks has decreased significantly in the past decades, more than 0.137 g_Pt_ kW^−1^ is still needed^5^. One promising approach to reduce the cost of fuel cells is to switch the operating environment from an acidic to a basic one (that is, a hydroxide exchange membrane fuel cell, HEMFC), thus opening up the possibility of using PGM-free catalysts and other cheaper components^6^. For the cathode of the HEMFC, some PGM-free and metal-free ORR catalysts have been developed that show comparable activity to Pt in alkaline media^7^,^8^,^9^,^10^. However, for the anode side, only a few PGMs (for example, Pt, Ir and Pd) show adequate activity^11^,^12^. The HOR catalysed by Pt is very fast in acidic conditions so that a very low loading of the Pt catalyst could be used relative to the cathode side in PEMFCs. However, the HOR activities of PGMs are ∼100 times slower in alkaline solutions^11^,^13^. As a result, a much higher loading of the HOR catalyst is required (0.4 mg_Pt_ cm^−2^ in a HEMFC compared with 0.03 mg_Pt_ cm^−2^ in a PEMFC) to achieve similar performance^5^,^14^. Thus, it is highly desirable to develop PGM-free anode catalysts for the HOR in alkaline electrolyte.

Unlike its reverse reaction (hydrogen evolution reaction, HER)^15^,^16^,^17^, only a few PGM-free HOR catalysts have been reported. One possibility is to use Raney Ni as the HOR catalyst in liquid alkaline fuel cells^18^,^19^,^20^. However, it is functional only under very high alkalinity (6 M KOH) while the activity remains low. It is not catalytically active for a HEMFC, which can be mimicked as 0.1–1 M KOH^21^. Efforts have been made to improve the HOR activity of the Ni-based catalyst in the last decade. Ni alloys, such as NiMo and NiTi, have been shown to enhance the HOR activity^20^. Our recent work has also shown that electrochemically deposited NiCoMo on an Au substrate has a high HOR activity^22^. Zhuang and co-workers decorated Ni particles with CrO_x_ to weaken the Ni–O bond and stabilize the Ni catalysts. A HEMFC incorporating this PGM-free catalyst has been fabricated, and it exhibits a peak power density of 50 mW cm^−2^ (ref. 21) Although the power density is still low (compared with the peak power density of more than 1,000 mW cm^−2^ for PEMFCs), it demonstrates the possibility to fabricate low-cost PGM-free fuel cells. However, their activities are still incomparable with PGM-based catalysts.

In the current study, we synthesize Ni nanoparticles supported on N-doped carbon nanotubes (Ni/N-CNT) by a wet chemical method, and the Ni/N-CNT shows a high HOR catalytic activity in 0.1 M KOH solution. N-CNTs are not only the support for the Ni nanoparticles, but also a promoter for the catalytic activity. Owing to its special electronic properties, the N-doped carbon structure has been employed to enhance the catalytic activity of the methanol oxidation reaction and the ORR^23^. We use N-CNT as a support for the HOR catalyst and demonstrate that, relative to Ni nanoparticles, the mass activity and exchange current density of Ni/N-CNT increases by a factor of 33 and 21, respectively. To understand the interaction between the Ni nanoparticle and the N-CNT support, density functional theory (DFT) calculations are undertaken. The DFT calculations indicate that, when nitrogen dopants are present at the edge of the nanoparticle, the Ni nanoparticle is stabilized on the support and locally activated for the HOR because of modulation of the Ni *d*-orbitals. Using the volcano relationship between the HOR activity and the hydrogen adsorption energy, predicted first-principles exchange current densities of the model systems are in good agreement with the measured exchange current densities of the experimental catalysts. Owing to its high activity and low cost, Ni/N-CNT has great potential to be used as the anode in HEMFCs, thereby finally bringing to fruition a high-performance and low-cost PGM-free HEMFC.

## Results

### Catalyst synthesis and characterization

A two-step approach was developed to synthesize the Ni/N-CNT hybrid catalysts. First, Ni was selectively grown on mildly oxidized multiwalled CNTs by reducing nickel salt in an aqueous solution. Second, ammonia and hydrazine were added and then subjected to a hydrothermal treatment at 150 °C. This step led to better crystallization of the Ni nanoparticles, partial reduction of the oxidized CNTs and, more importantly, doping of nitrogen into the CNTs.

**Figure 1:**
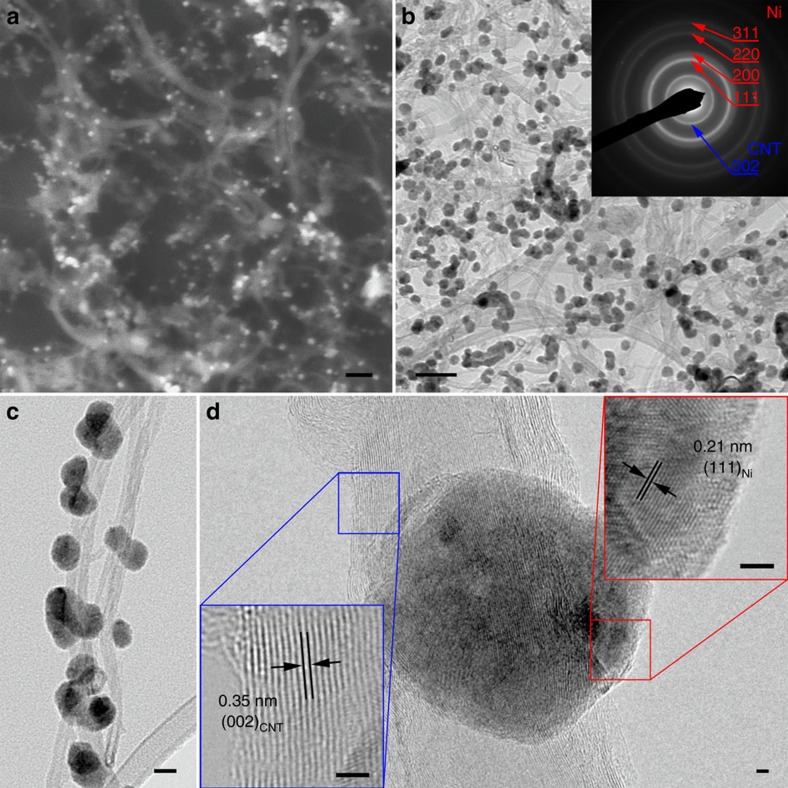
Electron microscopy of the Ni/N-CNT. (**a**) SEM image. Scale bar, 100 nm. (**b**) TEM image. Scale bar, 100 nm. Inset is selected area electron diffraction pattern. (**c**) A magnified TEM image. Scale bar, 20 nm. (**d**) HRTEM images of nickel particle and CNT, respectively. Scale bars, 2 nm.

The scanning electron microscopy (SEM, Fig. 1a) and transmission electron microscopy (TEM, Fig. 1b) images clearly show that the nanoparticles with an average size of 20 nm are selectively grown on the surface of the CNT. The diffraction rings in the selected area electron diffraction (inset of Fig. 1b) pattern can be either indexed to Ni or CNT. The energy-dispersive X-ray spectroscopy (EDS, Supplementary Fig. 1) also indicated the presence of the Ni component. The enlarged TEM image (Fig. 1c) shows that all the nanoparticles are directly supported on the CNT. Moreover, the high-resolution TEM (HRTEM, Fig. 1d) image clearly indicates the layered structure of the CNT wall, with an interplanar spacing of 0.35 nm, which is similar to the (002) plane of graphite. The lattice fringe of the nanoparticle has an interplanar spacing of 0.21 nm and is ascribed to the (111) plane of Ni.

**Figure 2:**
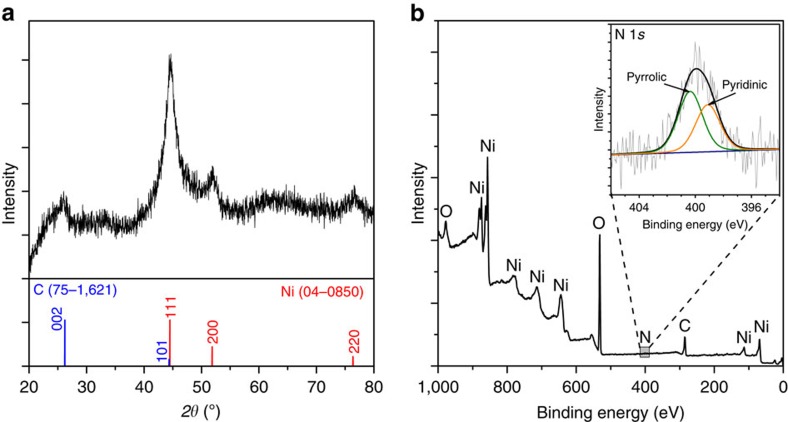
X-ray diffraction pattern and XPS spectra of the Ni/N-CNT. (**a**) X-ray diffraction pattern. The standard pattern of Ni (JCPDS card No. 04-0850) and graphite (JCPDS card No. 75-1621) are shown beneath the plot. (**b**) XPS spectrum. The inset is the high-resolution N 1*s* XPS spectrum.

The X-ray diffraction pattern (Fig. 2a) further confirms the mixed Ni and CNT composition of the catalyst. The loading of Ni on carbon was calculated as ∼70 wt % by thermogravimetric analysis (TGA, method shown in Supplementary Fig. 2). The N doping was examined with X-ray photoelectron spectroscopy (XPS, Fig. 2b). A peak at *ca*. 400 eV clearly exists in the high-resolution N 1*s* XPS spectrum (inset of Fig. 2b), confirming the N doping. It can be deconvoluted into two peaks, 399.0 and 400.3 eV, corresponding to pyridinic N and pyrrolic N in doped CNT, respectively^24^,^25^. The N-doping amount to carbon was calculated from the XPS spectrum as 2.4 at %.

### Electrochemical HOR performance

The electrocatalytic activity for the HOR was investigated by rotating disk electrode measurements using a standard three-electrode system in 0.1 M KOH. The catalyst was uniformly cast on a 5-mm glassy carbon electrode with a Ni loading of 0.25 mg_Ni_ cm^−2^. Undoped CNT supported Ni nanoparticles (represented as Ni/CNT) and unsupported Ni nanoparticles (represented as Ni) catalysts (corresponding TEM images and X-ray diffraction patterns are shown in Supplementary Figs 3 and 4, respectively) with the same loading were also studied for comparison.

**Figure 3:**
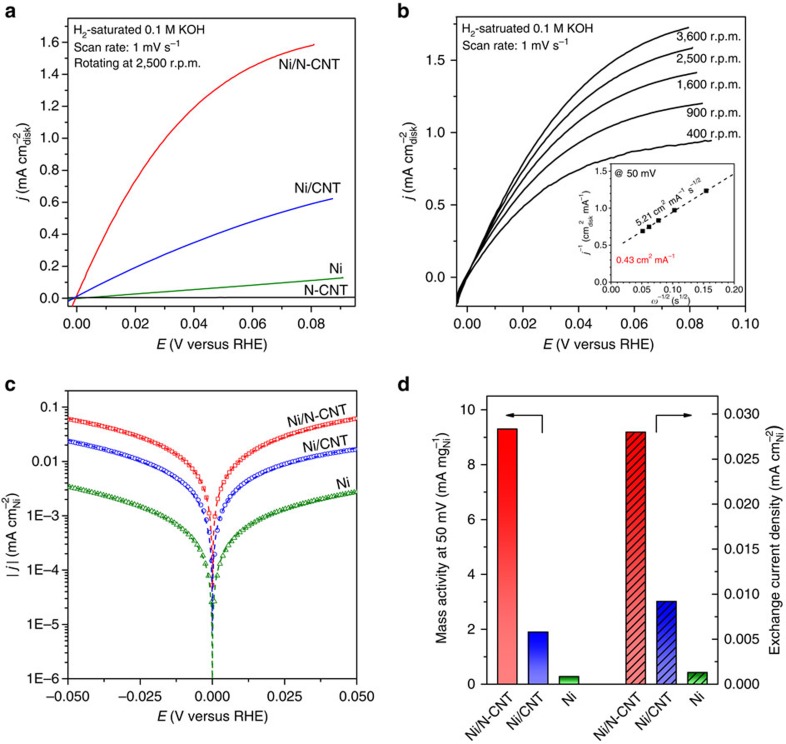
HOR performances. (**a**) Polarization curves of Ni/N-CNT, Ni/CNT, Ni (all of the three catalysts with a loading of 0.25 mg_Ni_ cm^−2^) and N-CNT (0.1 mg_C _cm^−2^) catalysts in H_2_-saturated 0.1 M KOH at a scan rate of 1 mV s^−1^ and rotating speed of 2,500 r.p.m. (**b**) Polarization curves of Ni/N-CNT in H_2_-saturated 0.1 M KOH at a scan rate of 1 mV s^−1^ and various rotating speeds. Inset is the Koutecky–Levich plot at an overpotential of 50 mV. (**c**) HOR/HER Tafel plots of the specific current density on Ni/N-CNT, Ni/CNT and Ni in H_2_-saturated 0.1 M KOH. The dashed lines indicate the Butler–Volmer fitting. (**d**) Mass activity at 50 mV (unpatterned) and exchange current density (patterned) of the Ni/N-CNT, Ni/CNT and Ni, respectively.

Figure 3a shows the polarization curve of the catalysts in H_2_-saturated electrolyte. The anodic current above 0 V (versus the reversible hydrogen electrode, same hereafter) is assigned to the oxidation of H_2_. The catalytic activity for HOR on the three Ni-based catalysts follows the sequence of Ni/N-CNT>Ni/CNT>Ni. The current density of Ni/N-CNT is much higher than either Ni/CNT or Ni and shows an onset potential as low as 0 V. The bare N-CNT shows almost no catalytic activity for HOR, indicating that the enhanced catalytic activity for Ni/N-CNT comes from the synergetic effect of the combined structure. The Ni/N-CNT catalyst was also tested in an Ar-saturated electrolyte (Supplementary Fig. 5) and showed no anodic current, confirming the catalytic reaction of HOR.

The polarization curves at different rotating speeds have also been studied (Fig. 3b). The current density increases with increasing rotating speed owing to improved mass transport. The overall current density is under mixed kinetic-diffusion control. The diffusion-limited current density (*j*_d_) for a rotating disk electrode is described by the Levich equation as a function of rotating speed (*ω*):


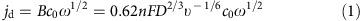


where *B* is the Levich constant, *c*_0_ is the solubility of H_2_ in 0.1 M KOH^26^. The Levich constant is a function of the diffusivity of H_2_ (*D*), number of the electrons (*n*) transferred in the HOR and kinematic viscosity of the electrolyte (*ν*). The overall current density (*j*) can be deconvoluted into kinetic (*j*_k_) and diffusional (*j*_d_) components following the Koutecky–Levich equation:


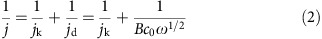


A linear relationship between the inverse of *j* at 50 mV and *ω*^1/2^ is observed in the Koutecky–Levich plots (inset of Fig. 3b). The calculated slope is 5.21 cm^2^ mA^−1^ s^−1/2^, which is reasonably close to the theoretical number (4.87 cm^2^ mA^−1^ s^−1/2^, for the two-electron transfer of HOR)^13^. The intercept of the extrapolated line corresponds to the inverse of the purely kinetic current density, and the *j*_k_ for Ni/N-CNT is 2.33 mA cm_disk_^−2^ at 50 mV overpotential. Normalizing by the metal loading of the catalyst, the mass activity of the Ni/N-CNT is 9.3 mA mg_Ni_^−1^ at 50 mV overpotential, shown in Fig. 3d and Supplementary Table 1 along with that of Ni/CNT and Ni. The mass activity of our synthesized unsupported Ni nanoparticles (0.28 mA mg_Ni_^−1^) is comparable to the activities of Raney Ni reported in the literature^27^,^28^,^29^,^30^,^31^,^32^. By contrast, the mass activity of the Ni/N-CNT is 33 times as high as the pure Ni nanoparticle, making Ni/N-CNT one of the most active PGM-free catalysts for HOR (Supplementary Table 2 lists the HOR mass activity of the PGM-free catalyst reported in the literature).

To understand the intrinsic activity of the Ni/N-CNT catalyst, we normalized the current by the electrochemical surface area (ECSA). The ECSAs of each Ni-based catalyst were measured in Ar-saturated 0.1 M KOH using a cyclic voltammetry method and were calculated from the OH desorption region of Ni subtracted by HER current using a charge density of 514 μC cm_Ni_^−2^ for one monolayer of OH adsorption on Ni (Supplementary Fig. 6)^33^,^34^. Figure 3c shows the ECSA-normalized kinetic currents as a function of the overpotential. The HOR kinetic current was obtained from the Koutecky–Levich equation (equation 2). The exchange current density (*j*_0_, shown in Fig. 3d and Supplementary Table 1) was obtained by fitting the ECSA-normalized HOR/HER kinetic current density to the Bulter–Volmer equation:


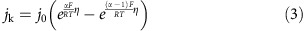


Here *α* is the charge transfer coefficient, *F* is Faraday's constant, *R* is the universal gas constant, *T* is the temperature and *η* is the overpotential^13^. All the curves can be fitted with a value of *α* in the range of 0.4∼0.5, indicating a good symmetry for the HOR and HER branches. The *j*_0_ values obtained from linear fitting of micropolarization regions (−10–10 mV, see Supplementary Fig. 7 and Supplementary Table 1) are consistent with the values of *j*_0_ obtained from Bulter–Volmer fitting. The Ni/N-CNT has an exchange current density of 0.028 mA cm_Ni_^−2^, which is three times higher than Ni/CNT and 21 times higher than Ni. The exchange current density for Ni/N-CNT is among the best PGM-free HOR catalysts and is comparable to some PGM catalysts, such as Pd (Supplementary Table 3)^11^,^35^. Although the HOR activity of the Ni/N-CNT is still over one order of magnitude less than the state-of-the-art Pt catalyst, the ultralow price of Ni makes it promising for commercial applications.

### Theoretical investigation

On the basis of a Wulff construction, fcc metals, such as Ni, are known to prefer a cuboctahedral geometry, exposing the {111} and {100} facets^36^. To model the nanoparticle, a 13-atom Ni nanocluster (Ni_13_) was chosen, as this was the smallest of the cuboctahedral ‘magic numbers' (a larger nanoparticle, Ni_37_, was also studied and is discussed below)^37^. Because of the relatively large size of both the Ni nanoparticles and carbon nanotubes used experimentally, previous studies suggest that the curvature of the CNT support has a minimal effect^38^; as such, the CNT support is approximated with a graphene sheet to reduce the complexity of the model. This results in a Ni_13_ nanoparticle being deposited on both graphene (Ni/graphene) and nitrogen-doped graphene (Ni/N-graphene), with both the Ni_13_ nanoparticle and the support being allowed to relax. We have investigated the effect of the location of the nitrogen dopant relative to the nanoparticle by considering nitrogen at the centre (N_c_) and nitrogen at the edge (N_e_), as shown in Supplementary Fig. 8.

In alkaline solutions, the HOR follows either the Tafel–Volmer or the Heyrovsky–Volmer mechanism. The possible elementary steps comprising the HOR are shown in Equations 4, 5, 6.


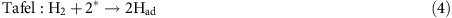



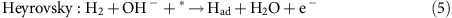



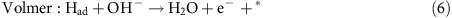


The key intermediate is the adsorbed hydrogen (H_ad_) on the catalyst surface, which leads to the existence of a Sabatier volcano relationship with the hydrogen-binding energy of the catalyst as the descriptor^39^. Although mechanistically the H_ad_ written in the elementary steps above is likely the overpotential deposited hydrogen (H_OPD_), the HOR/HER activity seems to correlate well to the electrochemical adsorption energy of the underpotential deposited hydrogen (H_UPD_), which is shown to be similar to the chemical adsorption energy of hydrogen^40^,^41^,^42^. In this study we calculate chemical adsorption energy of hydrogen on Ni_13_ nanoclusters.

The volcano relationship involving adsorbed hydrogen has been well established in acidic solutions^43^. While some debate remains on the adequacy of using the hydrogen-binding energy as a descriptor in alkaline media, there is significant evidence that the hydrogen-binding energy remains the key descriptor of activity^12^,^44^,^45^. This allows for the implementation of a hybrid data-driven/first-principles model to evaluate the efficacy of different catalysts and to estimate activity compared with single metals. This hybrid approach was employed successfully in propane total oxidation^46^. Details of the construction of the hybrid activity model are given in the Supplementary Methods.

**Figure 4:**
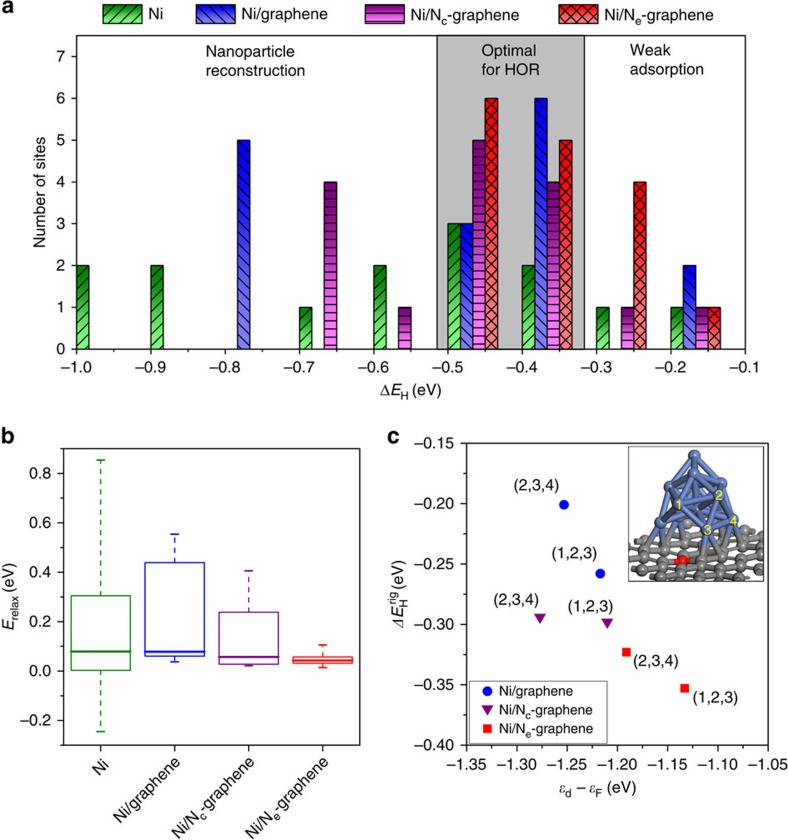
Computational results. (**a**) Distribution of site-dependent hydrogen-binding energies for each model system. (**b**) Distribution of relaxation energies for each model system on hydrogen-binding to each site. The ends of the dashed line represent the minimum and maximum values of *E*_relax_ for each model structure. These ranges collectively represent the distribution of *E*_relax_ values for each hydrogen adsorption site on the Ni_13_ cluster. The bottom of the box represents the first quartile (that is, splits the lowest 25% of relaxation energies from the highest 75%) and the top of the box represents the third quartile (that is, splits the highest 25% of relaxation energies from the lowest 75%). Bold horizontal band is the median value. (**c**) Shifts in the *d*-band centre with respect to the Fermi level and binding energy at adjacent Ni sites (1,2,3) and (2,3,4) for Ni/graphene, Ni/N_c_-graphene and Ni/N_e_-graphene. Inset is graphical depiction of the sites (Ni/N_e_-graphene as an example). The (1,2,3) site represents the hollow site in coordination with the #1, #2 and #3 Ni atoms. The (2,3,4) site represents the hollow site in coordination with the #2, #3 and #4 Ni atoms. Blue, grey and red spheres represent Ni, C and N atoms, respectively.

The heterogeneity of the hydrogen-binding sites in the supported Ni nanocluster model system results in a distribution of binding energies shown in Fig. 4a. Here we see a large amount of variability among the metal-support systems at high binding energies, with an unsupported Ni_13_ nanocluster and Ni/graphene having a large number of sites that adsorb hydrogen too strongly to be active for HOR. On the other hand, Fig. 4a shows that a Ni nanoparticle supported on N_e_-graphene does not possess large binding energies as in the unsupported Ni and the Ni/graphene systems.

The differences in the binding energies between the unsupported Ni_13_ nanocluster and the supported Ni_13_ arise from either an electronic (that is, charge transfer) or a geometric (that is, relaxation) origin^47^. For the strong binding sites, the nanocluster undergoes reconstruction as shown in Supplementary Fig. 9. The relative contributions of the electronic and the geometric support effects are decoupled by considering a nanocluster which is treated as rigid during hydrogen binding. For a rigid nanocluster, any differences in the binding energies result from the differences in the electronic structure of the Ni_13_ nanocluster because of the support. We define a relaxation energy, *E*_relax_, as


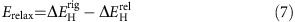


where Δ*E*_H_^ rig^ is the hydrogen-binding energy on the rigid nanocluster, and Δ*E*_H_^ rel^ is the hydrogen-binding energy on the relaxed nanocluster. Here a larger *E*_relax_ implies a larger degree of Ni reconstruction. Figure 4b shows *E*_relax_ for all Ni-binding sites for the systems considered. The unsupported nanocluster is the least stable during hydrogen binding. With the exception of a fraction of binding sites, the nitrogen-doped supports exhibit better stabilization of the Ni nanocluster. In agreement with its lack of strongly binding sites (*cf.* Fig. 4a), Ni/N_e_-graphene exhibits local relaxation with minimal reconstruction. In addition, when considering the rigid nanocluster, the difference in the average hydrogen-binding energy of Ni/N_c_-graphene and Ni/N_e_-graphene is less than 0.01 eV (versus 0.09 eV difference for the relaxed nanocluster). Thus, we observe that the rigid nanocluster's binding characteristics are not greatly affected by the support (N_c_-graphene or N_e_-graphene).

Next, we examine what fundamental electronic properties of the Ni_13_ nanocluster are being affected by the support. A commonly employed electronic descriptor for the binding energies on metal surfaces is the energy of the *d*-band centre, *ɛ*_d_, relative to the Fermi level, *ɛ*_F_ (ref. 48). The *d*-band centre describes the relative filling of the hybridized (*d*–*s*) and antibonding (*d–s*)* states of the Ni–H system. For an adsorbate, such as atomic hydrogen with a low-lying electronic state, the stabilization of the bonding states between the metal *d*-states and the hydrogen *s*-states decreases when the *d*-band of the metal is shifted up in energy (*ɛ*_d_–*ɛ*_F_ is more positive) because of the energy mismatch between the metal *d*-band and the hydrogen *s*-state. As a result, the resulting antibonding part of the band is pushed up just above the Fermi level, which leads to stronger binding between the metal atoms and the adsorbate. In Fig. 4c, we see an upshift in the *d*-band centre of adjacent Ni-binding sites when an edge nitrogen atom is present. Owing to these sites originally lying in the weak binding region of Fig. 4a, the upshift in the *d*-band centre consequently activates these sites adjacent to N_e_ for the HOR by strengthening the binding energy by 0.1 eV. XPS valence band spectra, which are related to the *d*-band centres, were also studied (Supplementary Fig. 10)^49^. Thus, while the geometric effect of the support is responsible for the decrease in strong binding sites, edge nitrogen further tunes local binding sites by modulating the electronic properties of Ni.

**Figure 5:**
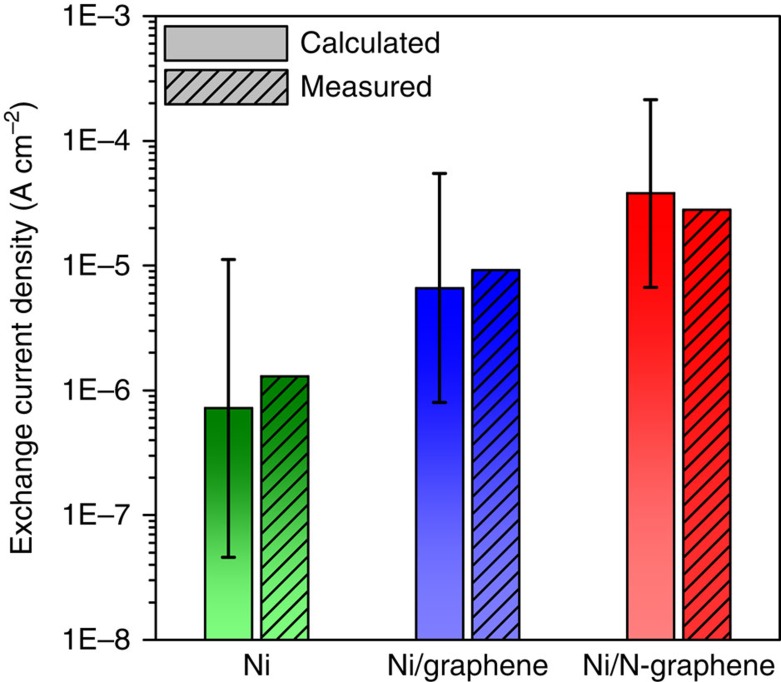
Comparison of calculated exchange current densities to measured values. Unpatterned bars are the calculated exchange current densities and patterned bars are the measured values. The calculated exchange current density of Ni/N_e_-graphene is shown for Ni/N-graphene. Error bars are 75% confidence intervals resulting from the regression of the volcano relationship in Supplementary Equation 2.

Using the hybrid activity model for the HOR, we can now predict the intrinsic exchange current density of each of the model systems that were investigated (Supplementary Fig. 11 and Supplementary Note 1). In agreement with experimental measurements, Fig. 5 shows that an order of magnitude increase in the exchange current density is predicted for a graphene-supported nanocluster compared with an unsupported Ni catalyst. Additional improvement occurs when doping the graphene support with nitrogen, where having nitrogen at the edge of nanocluster (that is, directly interacting with surface Ni atoms) appears to be more effective than having nitrogen at the centre of the nanoparticle. Relating this discovery to the current experimental procedure, it is expected that edge nitrogen is predominant in the experimental material. This is because nitrogen doping is performed after the Ni nanoparticle was grown from the CNT surface. Thus, it is expected that only sites at the edge of the nanoparticle are available for nitrogen doping, and that lattice diffusion is the only mechanism by which nitrogen could be transported to the centre of the nanoparticle.

To confirm that the Ni_13_ is sufficiently large to capture the metal-support interaction of the experimental system, we performed calculations on a larger Ni_37_ nanoparticle (Supplementary Note 2). The *d*-orbitals of the Ni_13_ cluster correspond well to the larger Ni_37_ model (Supplementary Fig. 12). The difference in the calculated *d*-band centre is only 0.01 eV for the two nanoparticles, which indicates that the Ni_13_ is a good approximation for larger nanoparticles both in terms of its adsorption and electronic properties. The electronic effect of the N-dopant was found localized to the adsorption sites of the nearby Ni atoms (Supplementary Fig. 13 and Supplementary Table 4). Our findings suggest that even higher HOR activity could be obtained by making smaller Ni nanoparticles because of a larger fraction of adsorption sites that could be affected electronically by the N-dopant.

In summary, Ni/N-CNT as a highly active PGM-free HOR catalyst in alkaline condition was reported in this paper. The promoted HOR activity is attributed to the synergetic effect of the edge N atom in the CNT and Ni. The theoretical investigation indicated that the Ni nanoparticle has more sites with an optimal hydrogen-binding energy because of both a geometric and electronic interaction with the edge N atoms. Relative to Ni nanoparticles, the mass activity and exchange current density of Ni/N-CNT increases by a factor of 33 and 21, respectively. These results show that Ni/N-CNT is promising to be applied as the anode catalyst for HEMFCs.

## Methods

### Synthesis of nickel-based composite catalyst

In a typical synthesis of Ni/N-CNT, mildly oxidized MWCNTs (25 mg, 10–20 nm in diameter, Shenzhen Nanotech Port Co. Ltd., mildly oxidized through the modified Hummers method^50^) were dispersed in deionized water (10 ml) assisted by sonication for 30 min. After that, NiSO_4_·6H_2_O (0.262 g, 1 mmol) was added to the MWCNT suspension and sonicated for another 30 min. The suspension was transferred to a flask placed in an ice-water bath. Then ice-cold NaBH_4_ aqueous solution (1 wt %, 10 ml, ∼2.5 mmol) was added dropwise. After reaction at 0 °C for 2 h, the suspension was transferred to a 45-ml Teflon-lined stainless steel autoclave. Ammonium hydroxide solution (28–30% NH_3_ basis, 5 ml) and hydrazine hydrate solution (78–82%, 2 ml) were added, and then the autoclave was sealed and subjected to a hydrothermal treatment at 150 °C for 4 h. The final product was collected by centrifugation at 8,000 r.p.m. for 10 min, washed with water and then redispersed in water (10 ml).

Ni and Ni/CNT were synthesized by similar procedures. Ni was synthesized without adding oxidized MWCNT and the pH was adjusted to 12 by ammonium hydroxide before the hydrothermal treatment. Ni/CNT was synthesized without adding ammonium hydroxide and hydrazine.

### Physical characterization

The SEM was performed on a JEOL JSM-7400F SEM working at an acceleration voltage of 3 kV. The TEM, HRTEM and EDS were performed on a JEOL JEM-3010 TEM operating at 300 kV equipped with EDS system. The X-ray diffraction patterns were collected on a Bruker D8 Discovery diffractometer with Cu Kα radiation (*λ*=0.15418, nm) operating at 40 kV and 40 mA. The TGA was performed on a Mettler Toledo TGA/DSA 1 STAR^e^ System under air flow (60 ml min^−1^) with a heating rate of 5 °C min^−1^. The XPS spectra were recorded on Thermo Fisher ESCALAB 250Xi XPS system with a monochromatic Al Kα X-ray source. The survey scans were conducted at a pass energy of 100 eV using a step size of 1 eV, and the high-revolution scans were conducted at a pass energy of 50 eV using a step size of 0.05 eV.

### Electrochemical tests

The electrodes were prepared by casting catalyst inks on glassy carbon electrodes (5 mm in diameter, Pine Instruments, polished to a mirror-finishing with 0.05 μm alumina). The catalyst inks were prepared by dispersing catalyst in water with 0.05 wt % Nafion at a concentration of 2.5 mg_Ni_ ml^−1^. The catalyst ink (20 μl) was deposited on a glassy carbon electrode and dried in vacuum at room temperature, resulted in a metal loading of 0.25 mg_Ni_ cm^−2^. Electrochemical studies were carried out in a standard three-electrode system controlled with a multichannel potentiostat (Princeton Applied Research). As-prepared thin film electrode, which served as the working electrode, was mounted on a rotator and was immersed into 0.1 M KOH solution. An Ag/AgCl electrode with double junctions was used as the reference electrode. A Pt wire was used as the counterelectrode, which has an outer glass tube with a ceramic fritz to prevent the contamination of Pt. All potentials reported in this paper are referenced to the reversible hydrogen electrode potential. All the polarization curves were corrected for solution resistance, which was measured using AC-impedance spectroscopy from 200 kHz to 100 mHz and a voltage perturbation of 10 mV.

### Computational methodology

Periodic DFT calculations were performed using the Vienna *ab initio* software package (VASP, version 5.3.2)^51^. The optPBE-vdW exchange-correlation functional^52^ was selected because of its qualitatively accurate representation of the potential energy surface of a Ni monolayer and graphene system^53^. The core electrons were represented with the projector-augmented wavefunction (PAW) method^54^,^55^, and a plane-wave cutoff of 400 eV was used for the valence electrons. The Methfessel–Paxton method of electron smearing was used with a smearing width of 0.1 eV (ref. 56). All geometry optimizations were performed using the conjugate gradient algorithm^57^ as implemented in VASP. The forces and energies were converged to 0.05 eV Å^−1^ and 10^−4^ eV, respectively. All calculations were spin-polarized because of the magnetic moment of Ni. For the Ni nanocluster systems, all calculations were performed at the Γ-point. A 5 × 5 graphene unit cell was used as the support. In the *z* direction, a vacuum layer of 15 Å was included. This unit cell was chosen to minimize interaction of the Ni_13_ nanocluster with its periodic image while still being computationally tractable. For the single metal surfaces used to establish the hybrid activity model, a 4 × 4 unit cell was used with a 3 × 3 × 1 Monkhorst–Pack *k*-point sampling of the Brillouin zone^58^.

## Additional information

**How to cite this article**: Zhuang, Z. *et al.* Nickel supported on nitrogen-doped carbon nanotubes as hydrogen oxidation reaction catalyst in alkaline electrolyte. *Nat. Commun.* 7:10141 doi: 10.1038/ncomms10141 (2016).

## Supplementary Material

Supplementary Information Supplementary Figures 1-13, Supplementary Tables 1-4, Supplementary Notes 1-2, Supplementary Methods and Supplementary References
