# Supplementary material for: Nickel supported on nitrogen-doped carbon nanotubes as hydrogen oxidation reaction catalyst in alkaline electrolyte
Source: Nat Commun. 2016 Jan 14;7:10141. doi: 10.1038/ncomms10141 (PMC4735558; doi:10.1038/ncomms10141)
Supplement: Supplementary Information — Supplementary Figures 1-13, Supplementary Tables 1-4, Supplementary Notes 1-2, Supplementary Methods and Supplementary References [file ncomms10141-s1.pdf]

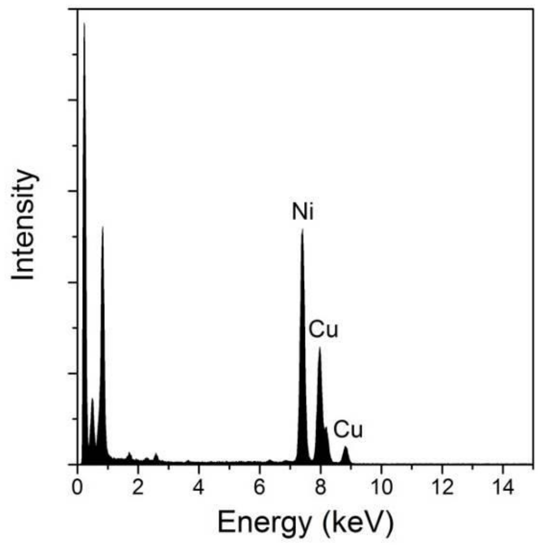

Supplementary Figure 1. EDS spectra of the Ni/N-CNT catalysts obtained on the JEOL JEM-3010 TEM equipped with EDS. The Cu peaks come from the copper grid.

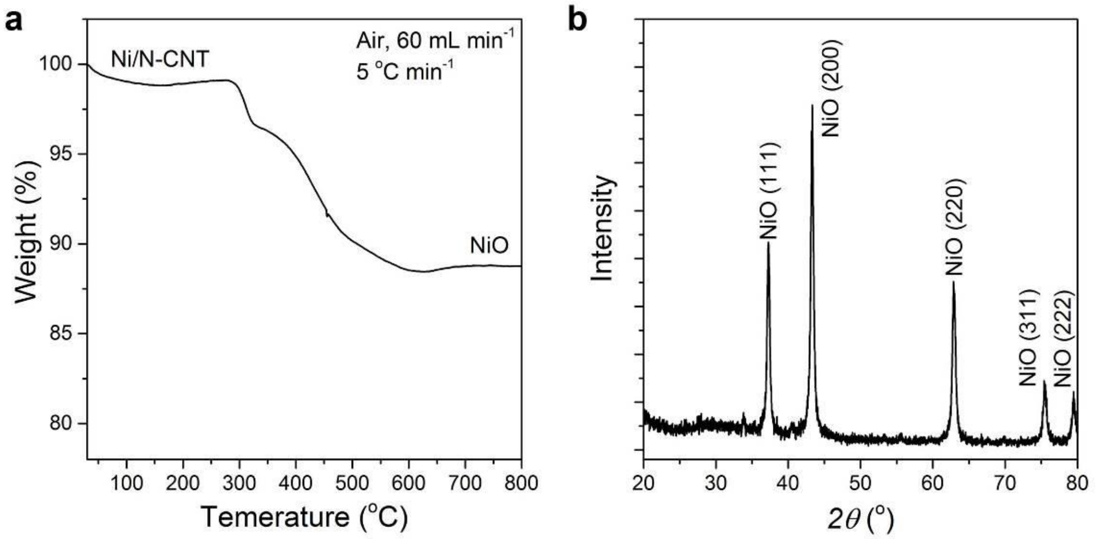

Supplementary Figure 2. (**a**) TGA curve of the Ni/N-CNT catalyst. (**b**) XRD pattern of the residue after TGA test. The TGA was performed under air atmosphere. N-CNTs were burned off and Ni was oxidized to NiO, which was confirmed by the XRD pattern (JCPDS card No. 47-1049). The weight remained after the TGA test is 88.7 wt %. Based on the formula of NiO, 78.6 wt % of NiO comes from Ni. Thus the Ni loading in Ni/N-CNT is 88.7 wt % ×78.6 wt % = 70 wt %.

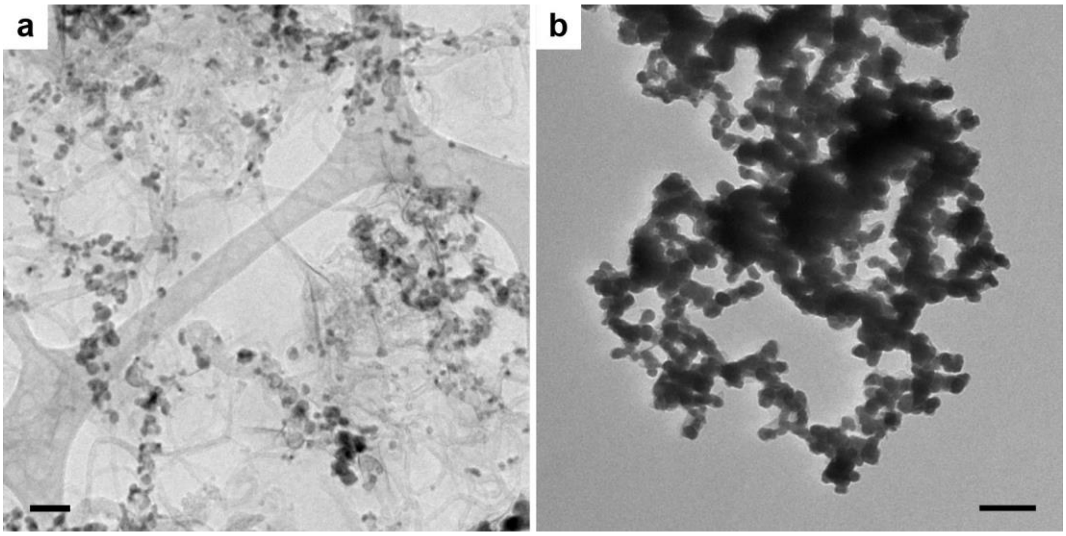

Supplementary Figure 3. TEM images of (**a**) Ni/CNT, (**b**) Ni. The scale bars, 100 nm.

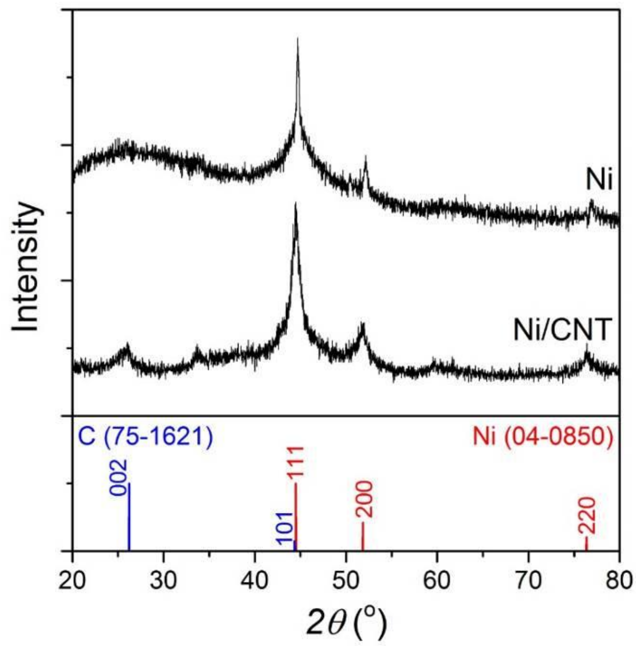

Supplementary Figure 4. XRD patterns of Ni/CNT and Ni. The standard pattern of Ni (JCPDS card No. 04-0850) and graphite (JCPDS card No. 75-1621) are shown beneath the plots.

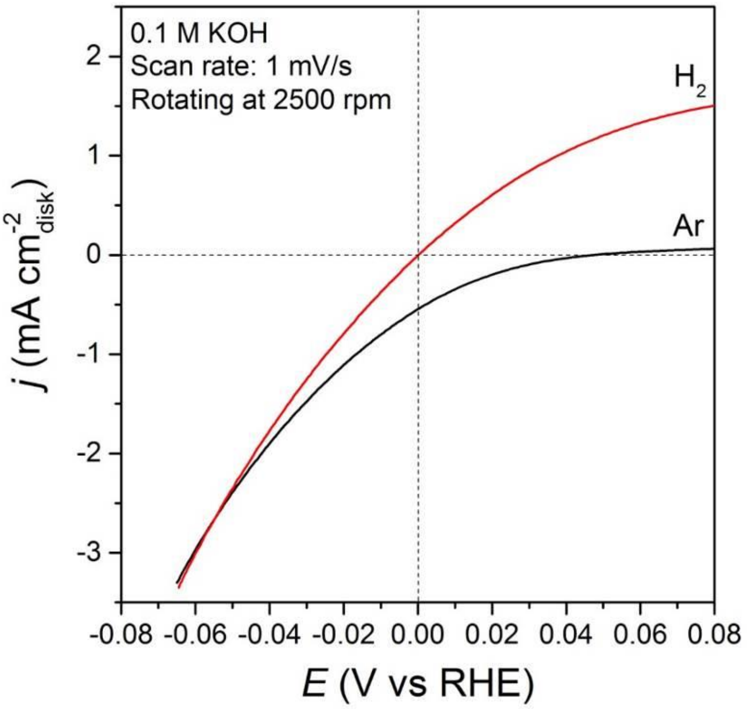

Supplementary Figure 5. Polarization curves of Ni/N-CNT in H_2 or Ar saturated 0.1 M KOH at a scan rate of 1 mV s^{-1} and rotating speed of 2500 rpm. To confirm the anodic current comes from HOR, we further tested the samples in an Ar-saturated solution. No anodic current was found when H_2 was replaced by Ar, indicating that H_2 is a reactant for the reaction. However, a cathodic current below 0 V was also observed in Ar atmosphere, which is similar in H_2 atmosphere. This cathodic current is attributed to the HER in which water is the reactant, and thus can be observed in both atmospheres.

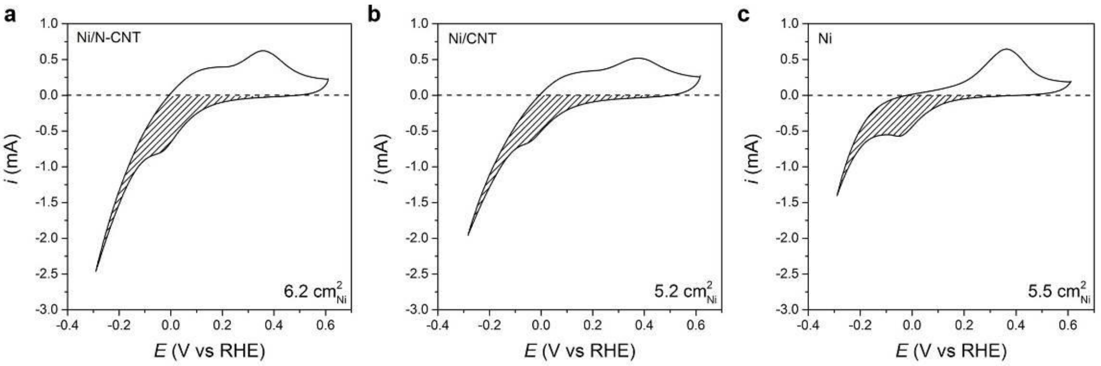

Supplementary Figure 6. Cyclic voltammetry (CV) of the catalysts in Ar-saturated 0.1 M KOH at a scan rate of 50 mV s^{-1}: (**a**) Ni/N-CNT, (**b**) Ni/CNT, (**c**) Ni. The patterned parts of the CV represent the area integrated to estimate the electrochemical surface area (ECSA) of Ni. The calculated ECSA of each catalyst are shown at lower right corner, respectively. The ECSA of Ni is calculated from the charge of the reduction of $Ni(OH)_2$ to Ni. The cathodic current at the backward scan not only comes from the reduction of $Ni(OH)_2$, but also captures hydrogen evolution current. We subtract the HER current, which is estimated from the cathodic current at the forward scan. Thus only the patterned areas were integrated, which is the charge from the reduction of $Ni(OH)_2$ without HER. The ECSAs were calculated from the integrated area using a charge density of 514 μC cm_{Ni}^{-2} for one monolayer of OH adsorption on Ni.

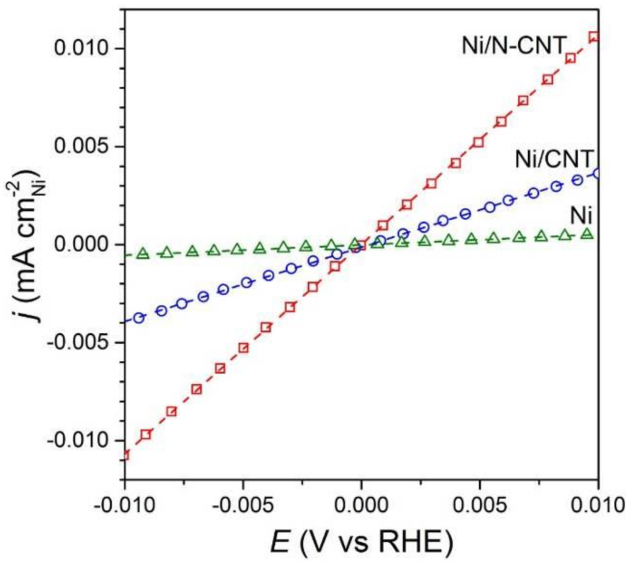

Supplementary Figure 7. Micro-polarization region (−10 mV to 10 mV) of Ni/N-CNT, N/CNT and Ni, respectively. The dash lines indicate the linear fitting.

In the micro-polarization region, the Bulter-Volmer equation can be simplified to

$$j_0 = \frac{j}{\eta}\frac{RT}{F} \tag{1}$$

where R is the universal gas constant, T is the temperature, F is Faraday's constant and j is the measured current density. Thus the exchange current density can be obtained from the slope of the linear fitting of j vs η. The obtained j_0 are listed in Supplementary Table 1, which are similar to the Bulter-Volmer fitting results.

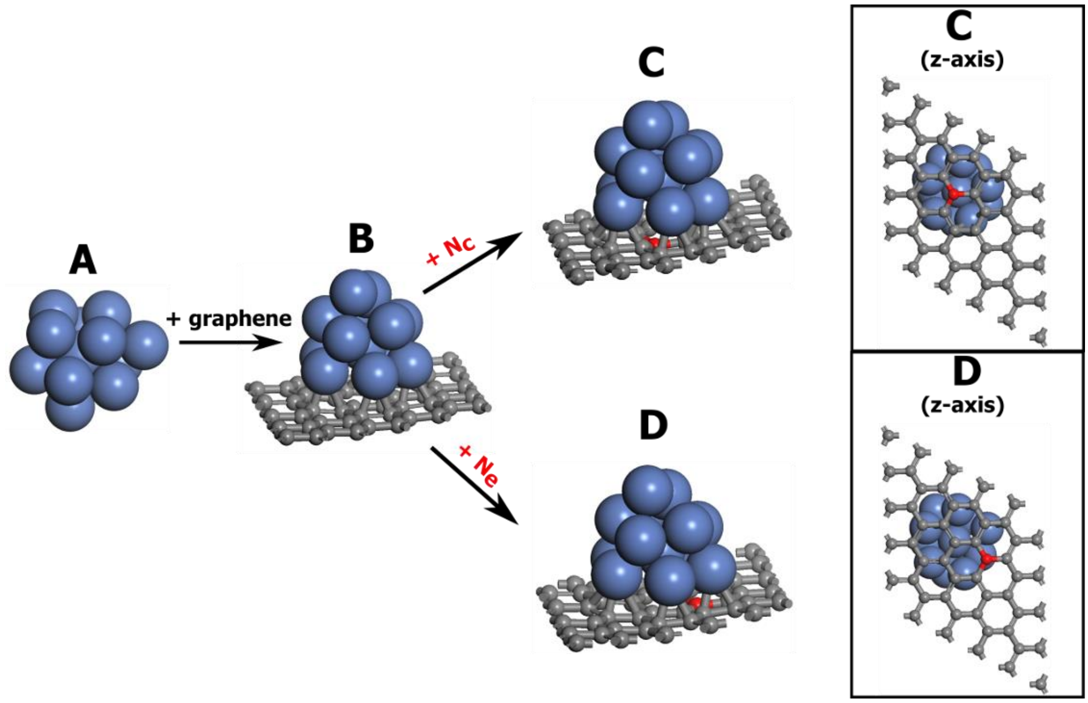

Supplementary Figure 8. Graphical depiction of the model systems considered in the current study. **A**: unsupported Ni_{13}, **B**: Ni/graphene, **C**: Ni/N_c-graphene, **D**: Ni/N_e-graphene (N_c and N_e are center and edge nitrogen, respectively). Inset shows viewpoint from the z-axis to visualize the nitrogen doping location. Blue, gray, and red spheres represent Ni, C, and N atoms, respectively.

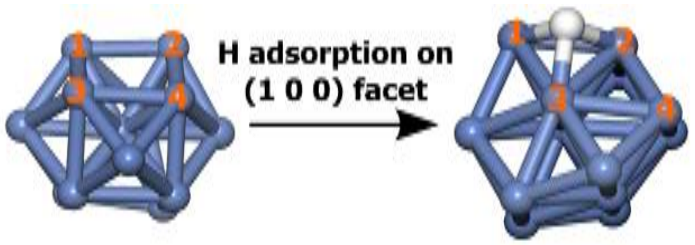

Supplementary Figure 9. Example of Ni reconstruction following hydrogen binding. Blue and white spheres represent Ni and H atoms, respectively.

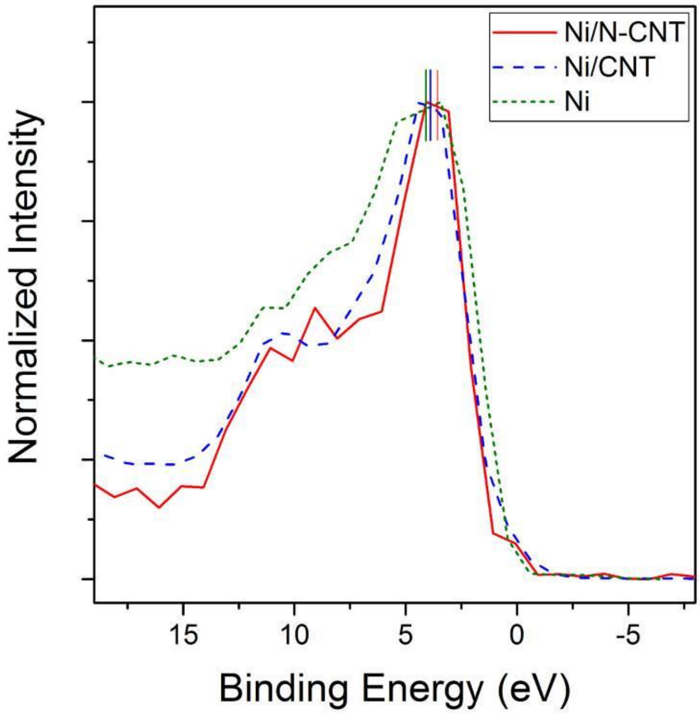

Supplementary Figure 10. Normalized XPS valence band spectra of Ni/N-CNT, Ni/CNT and Ni. The vertical lines represent the *d*-band centers. It can be observed that the valence band spectrum of Ni/N-CNT is narrower than those of Ni and Ni/CNT. The *d*-band center relative to the Fermi level follows the sequence of Ni (−4.0 eV) < Ni/CNT (−3.9 eV) < Ni/N-CNT(−3.7 eV), which is consistent with the DFT calculation results. The differences to the calculated *d*-band center shown in Figure 4c are mainly due to that the XPS measures the *d*-band of the whole nanoparticle, but only the *d*-band of the local sites affected by the N dopant calculated and shown in Figure 4c.

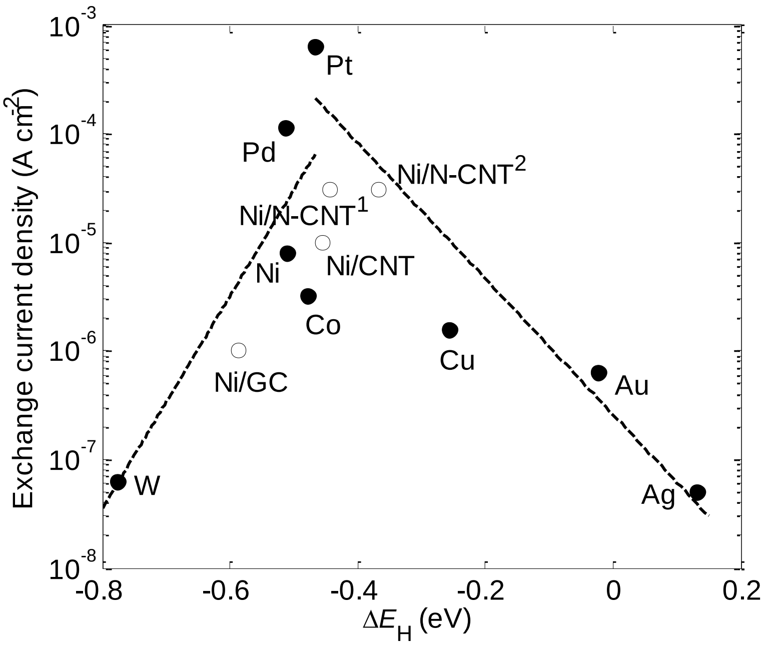

Supplementary Figure 11. Volcano plot showing *measured* exchange current densities as a function of the calculated hydrogen binding energy. Filled circles are single metal surfaces (polycrystalline disk, data from Ref 1) used in the regression to find the α and β parameters. Open circles represent the site-averaged binding energy of the nanocluster along with the measured activity of the experimental catalysts. The model systems are labeled according to their experimental counterparts, therefore: Ni/GC is unsupported Ni_{13}, Ni/CNT is Ni/graphene, Ni/N-CNT[1] is the Ni_{13} nanocluster supported on N_c-graphene, and Ni/N-CNT[2] is the Ni_{13} nanocluster supported on N_e-graphene. Fitting parameters for the ascending branch: α = 9.799 A cm^{-2} eV^{-1}, β = 0.384 A cm^{-2}; Fitting parameters for the descending branch: α = −6.254 A cm^{-2} eV^{-1}, β = −6.586 A cm^{-2}.

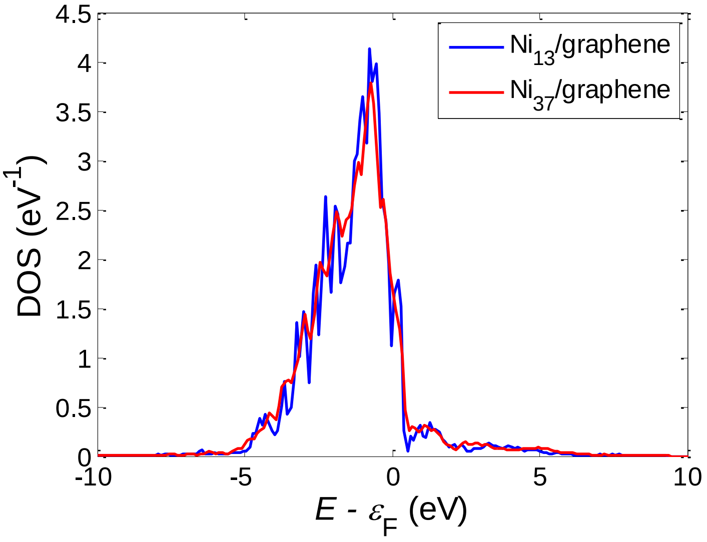

Supplementary Figure 12. Comparison of the *d* density of states (DOS) for the Ni_{13} and the Ni_{37} nanoparticles. The calculated *d*-band centers are −1.28 eV and −1.27 eV for the Ni_{13} and Ni_{37}, respectively. The Fermi level is the energy reference.

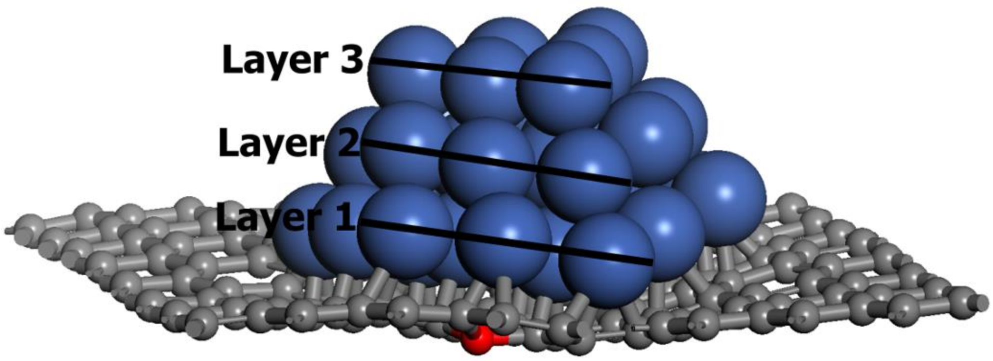

Supplementary Figure 13. Ni_{37} nanoparticle model supported on N-graphene with an edge nitrogen dopant. Blue, gray, and red spheres represent Ni, C, and N atoms, respectively.

Supplementary Table 1. Summary of the mass activity at 50 mV vs RHE and exchange current density of Ni/N-CNT, Ni/CNT and Ni. The exchange current densities are calculated by Bulter-Volmer fitting and micro-polarization methods.

Catalyst	Mass activity at 50 mV ($mA\ mg_{Ni}^{-1}$)	Exchange current density ($mA\ cm_{Ni}^{-2}$)	
		Bulter-Volmer fitting	Micro-polarization
Ni/N-CNT	9.3	0.028	0.027
Ni/CNT	1.9	0.0092	0.0097
Ni	0.28	0.0013	0.0013

Supplementary Table 2. Benchmark HOR mass activities of the PGM-free catalysts.

Catalyst	Electrolyte	Temperature (°C)	Mass activity at 50 mV ($mA\ mg^{-1}$)	Reference
Ni/N-CNT	0.1 M KOH	r. t.	9.3	This work
Ni/CNT	0.1 M KOH	r. t.	1.9	This work
Ni	0.1 M KOH	r. t.	0.28	This work
Raney Ni	6 M KOH	r. t.	0.15	2
Raney Ni	6 M KOH	23	0.11	3
Raney Ni/PTFE	25 wt % KOH (~5.5 M)	25	0.07	4
Raney-Ni/PTFE	25 wt % KOH (~5.5 M)	25	0.43	5
Raney Ni/PTFE	25 wt % KOH (~5.5 M)	25	0.27	6
Raney Ni/PTFE	30 wt % KOH (~6.9 M)	25	0.36	7
Ni-Mo	6 M KOH	23	1.8	6
Ni-Fe	6 M KOH	23	0.51	6
Ni-Ti	6 M KOH	23	1.5	6
Ni-Ti	6 M KOH	30	0.3	8
Ni-Mo	6 M KOH	30	5.3	9
Ni-W	6 M KOH	32	0.48	9
Raney Ni	6 M KOH	60	2.0	10
Raney Ni	6 M KOH	60	0.5	11
Ni-Ti	6 M KOH	60	1.4	11
Ni-Cr	6 M KOH	60	2.5	11
Ni-Cu	6 M KOH	60	0.9	11
Ni-Fe	6 M KOH	60	0.8	11
Ni-La	6 M KOH	60	2.0	11
Ni-Cr	6 M KOH	60	2.2	12
Ni-Ti	6 M KOH	60	1.8	12
Ni-Co	6 M KOH	80	4.0	13

Supplementary Table 3. Benchmark HOR exchange current densities of the PGM and PGM-free catalysts.

Catalyst	Electrolyte	Temperature (°C)	Exchange current density (mA cm_{metal}^{-2})	Reference
Ni/N-CNT	0.1 M KOH	r.t.	0.028	This work
Ni/CNT	0.1 M KOH	r.t.	0.0092	This work
Ni	0.1 M KOH	r.t.	0.0013	This work
NiCoMo	0.1 M KOH	r.t.	0.015	14
Pd/Au	0.1 M NaOH	20	0.023	15
Pt(pc)[a]	0.1 M NaOH	20	0.55	16
Pt(pc)[a]	0.1 M KOH	21	0.69	17
Pt/C	0.1 M KOH	21	0.57	17
Pt/C	0.1 M NaOH	40	1.0	18
Pd/C	0.1 M NaOH	40	0.06	18
Ir/C	0.1 M NaOH	40	0.37	18

[a]: pc = polycrystalline disk

Supplementary Table 4. Calculated *d*-band centers for undoped and doped cases for each layer of the Ni_{37} model. To explore the locality of the effect of the dopant, only the *d*-band centers of the three nearest neighbor Ni atoms for each layer shown above were taken into account.

Layer	Undoped: $\varepsilon_d - \varepsilon_F$ (eV)	Doped: $\varepsilon_d - \varepsilon_F$ (eV)	$\Delta(\varepsilon_d - \varepsilon_F)$ (eV)
1	−1.290	−1.233	+0.047
2	−1.271	−1.278	−0.007
3	−1.313	−1.315	−0.002

Supplementary Note 1

Calculation of the Exchange Current Density from the Volcano Plot. Supplementary Fig. 11 shows the volcano plot used to correlate the calculated, site-dependent hydrogen binding energies to exchange current density by obtaining the α and β parameters of Supplementary Equation 3. The regression in Supplementary Equation 3 was performed with respect to the close-packed surfaces of single metals (Ni, Pt, Co, etc.) for which exchange current density data was available (filled points in Supplementary Fig. 11).[1] For each of the threefold hollow sites on the four Ni_{13} model systems, the hydrogen binding energy was calculated from Supplementary Equation 2. In Supplementary Fig. 11, the open points representing the *site-averaged* binding energies for unsupported Ni, Ni/graphene, Ni/N_c-graphene, and Ni/N_e-graphene are shown with the experimentally measured exchange current densities of Ni/C, Ni/CNT, and Ni/N-CNT, respectively (note that no distinction is made experimentally between the N_c-graphene and N_e-graphene systems). Supplementary Fig. 11 indicates that binding energies between −0.3 and −0.5 eV were found to be optimal for HOR, in agreement with previous studies in acidic and alkaline solutions.[1, 19]

Supplementary Note 2

Calculations on a Larger Ni_{37} Nanoparticle. In order to evaluate the ability of the Ni_{13} model to capture the thermochemical and electronic properties of the large experimental nanoparticles, we compared the results with those on a Ni_{37} (truncated cuboctahedron) nanoparticle with a diameter of ~1.0 nm. The *d* density of states for the two models are compared in Supplementary Fig. 12 and the structural model of the Ni_{37} is shown in Supplementary Fig. 13. The *d*-orbitals of the Ni_{13} cluster correspond well to the larger Ni_{37} model. Importantly, the difference in the calculated *d*-band center is only 0.01 eV for the two nanoparticles. Activity predictions for the two models are also in close agreement: the exchange current density was predicted to be 6.7×10^{-3} mA cm^{-2} and 7.2×10^{-3} mA cm^{-2} for the Ni_{37}/graphene and the Ni_{13}/graphene, respectively.

Next we explore the electronic effect of the nitrogen-doped support of the N_e-graphene for the two nanoparticles. Supplementary Table 4, corresponding to the structural model in Supplementary Fig. 13, clearly shows that only the first layer of the Ni_{37} nanoparticle is significantly affected by the presence of the dopant. This provides evidence that the dopant has a *local electronic effect only* on the adsorption sites of the nearby Ni atoms. As a result, it is no surprise that the smaller Ni_{13} model exhibits a corresponding upshift in the *d*-band center with an edge nitrogen present.

Supplementary Methods

Hybrid Activity Model for Hydrogen Oxidation.

For each adsorption site *i*, the hydrogen binding energies are calculated according to Supplementary Equation 2,

$$\Delta E_{\mathrm{H}} = E_{\mathrm{metal+support+H}} - E_{\mathrm{metal+support}} - \frac{1}{2}E_{\mathrm{H_2}} \quad (2)$$

where $E_{\mathrm{metal+support+H}}$ is the total energy of the nanoparticle and support with a hydrogen atom adsorbed, $E_{\mathrm{metal+support}}$ is the energy of the nanoparticle and support, and $E_{\mathrm{H_2}}$ is the energy of a hydrogen molecule in the gas phase.

In order to estimate the overall exchange current density for a given model system, the ascending and descending branch of the volcano curve are correlated to the hydrogen binding energy at site i, $\Delta E_{\mathrm{H},i}$, via a linear scaling relationship with α and β as fitting parameters

$$\log j_{0,i}\ \left(\mathrm{mA\ cm^{-2}}\right) = \alpha \times \left(\Delta E_{\mathrm{H},i}\right) + \beta \quad (3)$$

Given the site heterogeneity of the Ni_{13} nanocluster, the local exchange current densities for each binding site are averaged according to the stochastic method of Gillespie.[20]

$$\overline{j_0} = \frac{1}{n}\sum_{i=1}^{n} j_{0,i} \quad (4)$$

In Supplementary Equation 3 and 4, $j_{0,i}$ is the exchange current density of binding site i as predicted by the volcano relationship given the hydrogen binding energy at that site, and n is the number of threefold Ni binding sites present on the nanoparticle surface, since atomic hydrogen preferentially binds on threefold hollow sites in our nanoparticle models.

Supplementary References

1. Sheng, W., Myint, M., Chen, J.G. & Yan, Y. Correlating the hydrogen evolution reaction activity in alkaline electrolytes with the hydrogen binding energy on monometallic surfaces. *Energy & Environmental Science* **6**, 1509-1512 (2013).
2. Ewe, H., Justi, E. & Schmitt, A. Studies of preservation of Raney-nickel-mixed catalysts. *Energy Conversion* **14**, 35-41 (1975).
3. Ewe, H., Justi, E. & Schmitt, A. Structure and properties of Raney-nickel catalysts with alloying for alkaline fuel-cells. *Electrochimica Acta* **19**, 799-808 (1974).
4. AlSaleh, M.A., Gultekin, S., AlZakri, A.S. & Khan, A.A.A. Steady state performance of copper impregnated Ni/PTFE gas diffusion electrode in alkaline fuel cell. *International Journal of Hydrogen Energy* **21**, 657-661 (1996).
5. SleemUrRahman, M.A., AlSaleh, M.A., AlZakri, A.S. & Gultekin, S. Preparation of Raney-Ni gas diffusion electrode by filtration method for alkaline fuel cells. *Journal of Applied Electrochemistry* **27**, 215-220 (1997).
6. Al-Saleh, M.A., Sleem Ur, R., Kareemuddin, S. & Al-Zakri, A.S. Novel methods of stabilization of Raney-Nickel catalyst for fuel-cell electrodes. *Journal of Power Sources* **72**, 159-164 (1998).
7. Jenseit, W., Khalil, A. & Wendt, H. Meterial properties and processing in the production of fuel-cell components. 1. Hydrogen anodes from Raney-nickel for lighweight alkaline fuel-cells. *Journal of Applied Electrochemistry* **20**, 893-900 (1990).
8. Mund, K., Richter, G. & Sturm, F.V. Titanium-containing Raney-nickel catalyst for hydrogen electrodes in alkaline fuel-cell systems. *Journal of the Electrochemical Society* **124**, 1-6 (1977).
9. Raj, I.A. & Vasu, K.I. Transision metal-based hydrogen electrodes in alkaline-solution-electrocatalysis on nickel based binary alloy coatings. *Journal of Applied Electrochemistry* **20**, 32-38 (1990).
10. Linnekoski, J.A., Krause, A.O.I., Keskinen, J., Lamminen, J. & Anttila, T. Processing of Raney-nickel catalysts for alkaline fuel cell applications. *Journal of Fuel Cell Science and Technology* **4**, 45-48 (2007).
11. Kiros, Y., Majari, M. & Nissinen, T.A. Effect and characterization of dopants to Raney nickel for hydrogen oxidation. *Journal of Alloys and Compounds* **360**, 279-285 (2003).
12. Kenjo, T. Doping effects of transition-metals on the polarization characteristics in Raney-nickel hydrogen electrodes. *Electrochimica Acta* **33**, 41-46 (1988).
13. Lee, H.K., Jung, E.E. & Lee, J.S. Enhancement of catalytic activity of Raney nickel by cobalt addition. *Materials Chemistry and Physics* **55**, 89-93 (1998).
14. Sheng, W., Bivens, A.P., Myint, M., Zhuang, Z., Forest, R.V., Fang, Q., Chen, J.G. & Yan, Y. Non-precious metal electrocatalysts with high activity for hydrogen oxidation reaction in alkaline electrolytes. *Energy & Environmental Science* **7**, 1719-1724 (2014).
15. Henning, S., Herranz, J. & Gasteiger, H.A. Bulk-Palladium and Palladium-on-

Gold Electrocatalysts for the Oxidation of Hydrogen in Alkaline Electrolyte. *Journal of the Electrochemical Society* **162**, F178-F189 (2015).

16. Rheinlaender, P.J., Herranz, J., Durst, J. & Gasteiger, H.A. Kinetics of the Hydrogen Oxidation/Evolution Reaction on Polycrystalline Platinum in Alkaline Electrolyte Reaction Order with Respect to Hydrogen Pressure. *Journal of the Electrochemical Society* **161**, F1448-F1457 (2014).
17. Sheng, W., Gasteiger, H.A. & Shao-Horn, Y. Hydrogen Oxidation and Evolution Reaction Kinetics on Platinum: Acid vs Alkaline Electrolytes. *Journal of the Electrochemical Society* **157**, B1529-B1536 (2010).
18. Durst, J., Siebel, A., Simon, C., Hasche, F., Herranz, J. & Gasteiger, H.A. New insights into the electrochemical hydrogen oxidation and evolution reaction mechanism. *Energy & Environmental Science* **7**, 2255-2260 (2014).
19. Norskov, J.K., Bligaard, T., Logadottir, A., Kitchin, J.R., Chen, J.G. & Pandelov, S. Trends in the exchange current for hydrogen evolution. *Journal of the Electrochemical Society* **152**, J23-J26 (2005).

20 Gillespie, D. T. A general method for numerically simulating the stochastic time evolution of coupled chemical reactions. *Journal of Computational Physics* **22**, 403-434 (1976).
